# Bayesian Inference on Proportional Elections

**DOI:** 10.1371/journal.pone.0116924

**Published:** 2015-03-18

**Authors:** Gabriel Hideki Vatanabe Brunello, Eduardo Yoshio Nakano

**Affiliations:** Department of Statistics, University of Brasília, Campus Darcy Ribeiro, Brasília-DF, Brazil; Tianjin University, CHINA

## Abstract

Polls for majoritarian voting systems usually show estimates of the percentage of votes for each candidate. However, proportional vote systems do not necessarily guarantee the candidate with the most percentage of votes will be elected. Thus, traditional methods used in majoritarian elections cannot be applied on proportional elections. In this context, the purpose of this paper was to perform a Bayesian inference on proportional elections considering the Brazilian system of seats distribution. More specifically, a methodology to answer the probability that a given party will have representation on the chamber of deputies was developed. Inferences were made on a Bayesian scenario using the Monte Carlo simulation technique, and the developed methodology was applied on data from the Brazilian elections for Members of the Legislative Assembly and Federal Chamber of Deputies in 2010. A performance rate was also presented to evaluate the efficiency of the methodology. Calculations and simulations were carried out using the free R statistical software.

## INTRODUCTION

In Brazil, elections for president, governors and mayors use the majority system, where the candidate with absolute majority of the votes is elected. On a proportional system however, the absolute majority of the votes do not guarantee the election of this candidate. The proportional scenario is the kind of election that deputies (federal, state and district) as well as members of the city council are elected from. A problem with proportional elections is the difficulty to evaluate the precise number of seats (vacancy) that each party won. Since there is no guarantee that the ratio between the number of votes and the number of seats is an integer, an approximation and redistribution system must take place. Brazil defines the electoral quotient as the number of valid votes divided by the number of seats. Each party has its votes divided by the electoral quotient to obtain the party quotient, and the integer part of this quotient corresponds to the number of seats reserved to the party. The remaining seats are then allocated using the D’Hondt method. These peculiarities of proportional elections make classic statistical inference not viable. However, the same inference can be easily carried out using Bayesian inference combined with Monte Carlo simulation methods. In this context, the purpose of this paper was to perform a Bayesian inference on proportional elections considering the Brazilian system of seats distribution. More specifically, a methodology to answer the probability that a given party will have representation (at least one seat) on the chamber of deputies was developed. Inferences were made on a Bayesian scenario using the Monte Carlo simulation technique and calculations and simulations were carried out using the R software. The developed methodology was applied on data from the Brazilian election for Members of the Legislative Assembly and Federal Chamber of Deputies in 2010.

## METHODS

### Brazilian Proportional Election System

The proportional election is an electoral system in which the proportion of taken seats of each party is determined by the proportion of obtained votes. It is utilized with the intention of ensuring the participation of different segments of society, because unlike the majority system, proportional elections do not necessarily guarantee the candidate with the most number of votes will be elected. In Brazil, elections for Federal Deputies, Members of the Legislative Assembly and Councilor’s use the proportional system.

The seat distribution is accomplished using the electoral quotient and the D’Hondt method for the distribution of the remaining seats [1,2]. The electoral quotient is the sum of all valid votes (nominal votes + party votes, which is equivalent to the total of votes minus the blank and null votes) divided by the number of available seats. Only parties (or coalitions) with a total of valid votes greater than the electoral quotient will participate on the D’Hondt method.

Initially, parties with a total of votes greater than the quotient will earn an amount of seats equal to the number of votes the party has divided by the quotient. In case of decimals, the value is rounded down. After the distribution, the remaining seats are distributed using the D’Hondt method, where the party with greatest number of adjusted votes (party’s votes divided by the number of earned seats plus 1) earns one more seat and has its total of votes readjusted. This procedure is used until there are no empty seats.

### Seats division method

The algorithm used for the division of seats on the Brazilian proportional electoral system is presented below [1,2].

**Step 0:** Get the data of the parties’ names, number of votes for each party and the number of available seats;
**Step 1:** Sum the number of valid votes (total of votes discarding null and blank votes) and divide by the number of seats. This result is the **electoral quotien**t;
**Note:** If no party receives more votes than the electoral quotient, the election is cancelled (no party earns any seats);
**Step 2:** Divide the number of each party votes by the electoral quotient and for each party, add a number of seats equal to the number gotten rounded down;
**Step 3:** If there are no remaining seats after the division by the quotient, the distribution is done and display the quantity of seats that each party (or coalition) earned;
**Step 4:** If there are remaining seats after Step 2, distribute them using D’Hondt method:
**Step 4.1:** To identify the party with the most adjusted votes, where
$\mathrm{Adjusted Votes}=\frac{\mathrm{party valid votes}}{\mathrm{earned seats}+1}$
**Note:** In case of a draw between two or more parties on the number of adjusted votes, the one with the smallest number of earned seats gets the seat.
**Step 4.2:** Add a seat to the party with the greatest number of adjusted votes in Step 4.1;
**Step 4.3:** If the number of remaining seats is greater than 0, return to Step 4.1, else, the distribution is complete.

### Bayesian Inference

Initially, a Bayesian analysis was done to the proportion of votes received by each party/coalition. This analysis was made through Dirichlet-Multinomial conjugation [3].

**Dirichlet-Multinomial Conjugation.** *Let X*_1_,…,*X*_*n*_ *be a random sample of size n*, *where X*_*j*_ = (*X*_1*j*_, …, *X*_*kj*_), *j =* 1,…,*n has a Multinomial distribution with parameters vector* (*θ*_1_, …,*θ*_*k*_), 0≤θ_*1*_ *≤*1, *and $\sum_{i=1}^{k}\theta_{i}=1$. Assume that the prior distribution of* (*θ*_1_, …,*θ*_*k*_) *is a Dirichlet with known hiper-parameters* (*a*_1_, …,*a*_*k*_), *a*_*i*_ *>*0, *∀ i =* 1,…,*k*. *Thus*, *the posterior distribution of* (*θ*_1_, …,*θ*_*k*_) *given X*_*j*_ *= x*_*j*_, *j =* 1,…,*n is a Dirichlet with parameters vector* (*a*_1_ *+y*_1_, …,*a*_*k*_ *+y*_*k*_), *where $y_{i}=\sum_{j=1}^{n}x_{ij}$, i* = 1,…,*k*.

Assume that the opinion of each elector is independent and, that in a specific moment, each one of them may: to opt for one of the *k* parties/coalitions; or to opt for a blank/null vote or even be indecisive. We will assume that indecisive voters are not informative, being excluded from the sample (notice that this procedure is different from assuming that they may opt for one of the *k* parties with same probability). Let *Y*_*j*_ be the number of voters favorable to the party *j*, *j* = 1,2,…,*k*, and *Y*_*k*+1_ the number of voters that pretend to vote blank/null. Selected a sample, the likelihood function of the data is given by:

$$L(Y_{1},...,Y_{k},Y_{k+1}|\theta_{1},...,,\theta_{k},\theta_{k+1})\propto \prod_{j=1}^{k+1}\theta_{j}^{y_{j}}$$

where $n=\sum_{j=1}^{k+1}y_{j}$ is the number of voters in the sample; *θ*_*j*_ is the true proportion of voters favorable to the party *j*, *j* = 1,2,…,*k* and *θ*_*k+*1_ is the true proportion of voters that pretend to vote blank/null. By the results of the Dirichlet-Multinomial conjugation, if a Dirichlet distribution with parameters vector (*a*_1_,…,*a*_*k*_,*a*_*k*+1_) is adopted as prior distribution, the posterior distribution of (*θ*_1_,…,*θ*_*k*_,*θ*_*k*+1_) given (*Y*_1_,…,*Y*_*k*_,*Y*_*k+*1_) is a Dirichlet with parameters vector (*a*_1_ *+y*_1_,…,*a*_*k*_ *+y*_*k*_,*a*_*k*+1_ *+y*_*k*+1_), i.e.,

$$\pi (\theta_{1},...,\theta_{k},\theta_{k+1}|Y_{1},...,Y_{k},Y_{k+1})=\frac{\Gamma \left(n+\sum_{j=1}^{k+1}a_{j}\right)}{\prod_{j=1}^{k+1}\Gamma (a_{j}+y_{j})}\prod_{j=1}^{k+1}\theta_{j}^{a_{j}+y_{j}-1}$$ 1

where $n=\sum_{j=1}^{k+1}y_{j}$ and $\Gamma (z)=\int_{0}^{\infty }t^{z-1}e^{-t}dt$ is the gamma function.

In a Bayesian scenario, the number of seats that each party earns is a multidimensional random variable and all information about this random variable is contained in its posterior density, whose analytic expression is unknown. However, it is not necessary to know the analytical form of the density of the seats, because its posterior can be easily obtained through Monte Carlo simulations methods [4]. The procedure consists in producing, from the posterior distribution of the proportion of votes (1), a large number of artificial elections and, in each one of them, to perform the seats distribution method described in the preceding section. Therefore, the probability of a determined party earning *c* seats is the number of times this party won *c* number of seats divided by the total of realized simulations.

### Performance Rate

To evaluate the efficiency of the methodology, a performance rate was developed. This rate ranges from 0 to 1, where 1 is a perfect score meaning that all the parties/coalitions got probability 1 on the number of seats they earned on the real election, and 0 is the opposite result, where the probability of each party earn the amount they earned on the real election is 0. The performance rate is calculated from the sum of the probability of each party earning the number of seats it obtained on the real election, divided by the number of parties/coalitions.

## RESULTS

### Election of MLA (Members of the Legislative Assembly)

The candidating parties to the election of the Members of the Legislative Assembly (MLA) in Federal District of Brazil in 2010 were: DEM (*Democratas*; Democrats), PCB (*Partido Comunista Brasileiro*; Brazilian Communist Party), PCO (*Partido da Causa Operária*; Workers Cause Party), PDT (*Partido Democrático Trabalhista*; Democratic Labor Party), PMDB (*Partido do Movimento Democrático Brasileiro*; Brazilian Democratic Movement Party), PP (*Partido Progressista*; Progressive Party), PMN (*Partido da Mobilização Nacional*; Party of National Mobilization), PPS (*Partido Popular Socialista*; Popular Socialist Party), PHS (*Partido Humanista da Solidariedade*; Humanist Party of Solidarity), PR (*Partido da República*; Party of the Republic), PRB (*Partido Republicano Brasileiro*; Brazilian Republican Party), PTB (*Partido Trabalhista Brasileiro*; Brazilian Labor Party), PSB (*Partido Socialista Brasileiro*; Brazilian Socialist Party), PC do B (*Partido Comunista do Brasil*; Communist Party of Brazil), PSC (*Partido Social Cristão*; Social Christian Party), PRTB (*Partido Renovador Trabalhista Brasileiro*; Brazilian Labor Renewal Party), PSDB (*Partido da Social Democracia Brasileira*; Brazilian Social Democracy Party), PSDC (*Partido Social Democrata Cristão*; Christian Social Democratic Party), PT do B (*Partido Trabalhista do Brasil*; Labor Party of Brazil), PSL (*Partido Social Liberal*; Liberal Social Party), PTN (*Partido Trabalhista Nacional*; National Labor Party), PSOL (*Partido Socialismo e Liberdade*; Socialism and Freedom Party), PSTU (*Partido Socialista dos Trabalhadores Unificados*; Unified Socialist Workers’ Party), PT (*Partido dos Trabalhadores*; Workers’ Party), PTC (*Partido Trabalhista Cristão*; Christian Labor Party), PRP (*Partido Republicano Progressista*; Progressive Republicam Party) and PV (*Partido Verde*; Green Party), totalizing 19 parties/coalitions presented in Table 1.

**Table 1:** Number of valid votes (nominal votes + party votes) and number of seats earned by the party/coalition in the election of the MLA in Federal District of Brazil, 2010.

**Parties/Coalitions**	**Valid Votes**	**Seats**	**Parties/Coalitions**	**Valid Votes**	**Seats**
DEM	102,840	2	PSC / PRTB	95,243	2
PCB	656	0	PSDB	84,044	1
PCO	270	0	PSDC / PT do B	80,141	1
PDT	76,814	1	PSL / PTN	83,571	1
PMDB	86,577	2	PSOL	25,439	0
PP / PMN	87,430	2	PSTU	966	0
PPS / PHS	109,126	2	PT	216,382	5
PR	70,175	1	PTC / PRP	73,124	1
PRB / PTB	96,548	2	PV	52,934	0
PSB / PC do B	83,381	1	Blank/Null/Missing	408,281	-

Source: Statistics of Brazilian Superior Electoral Court [5]

The 2010 election of MLA in Federal District of Brazil had 1,425,661 valid votes of 1,833,942 effective voters, and 24 empty seats were disputed between the parties/coalitions [5]. Inference were made using a sample of size *n* = 1000, randomly selected among effective voters.

To select the sample, it was considered the votes and parties shown on Table 1, including blank/null/missing and using R free software [6]. The sampling method was a simple random sampling with no replacement.

The probability of each party obtaining a quantity of seats was estimated adopting a non-informative prior *Dirichlet*(1,1,…,1) and 1,000,000 Monte Carlo simulations.

Table 2 presents the estimated probabilities (highlighting the real number of seats received by each party), the number of votes each party earned in the sample and the number of votes each party should earn in case of a perfect sample (a sample that describes the population perfectly).

**Table 2:** Estimated probabilities of obtaining seats for each party/coalition using a sample of 1,000 voters.

	Seats*									
Parties/Coalitions	0	1	2	3	4	5	6	7	N. of votes Normal Sample	N. of votes Perfect Sample
DEM	0.000	0.164	**0.817**	0.019	0.000	0	0	0	55	56
PCB	**1**	0	0	0	0	0	0	0	0	0
PCO	**1**	0	0	0	0	0	0	0	0	0
PDT	0.036	**0.690**	0.273	0.000	0	0	0	0	44	42
PMDB	0.000	0.085	**0.872**	0.044	0.000	0	0	0	58	47
PP / PMN	0.012	0.550	**0.437**	0.001	0	0	0	0	47	48
PPS / PHS	0.000	0.084	**0.872**	0.044	0.000	0	0	0	58	60
PR	0.384	**0.595**	0.021	0.000	0	0	0	0	35	38
PRB / PTB	0.000	0.039	**0.872**	0.089	0.000	0	0	0	61	53
PSB / PC do B	0.003	**0.387**	0.607	0.003	0	0	0	0	50	45
PSC / PRTB	0.008	0.497	**0.495**	0.001	0	0	0	0	48	52
PSDB	0.012	**0.551**	0.436	0.001	0	0	0	0	47	46
PSDC / PT do B	0.000	**0.200**	0.786	0.014	0.000	0	0	0	54	44
PSL / PTN	0.052	**0.725**	0.223	0.000	0	0	0	0	43	45
PSOL	**0.999**	0.000	0	0	0	0	0	0	12	14
PSTU	**1**	0	0	0	0	0	0	0	0	1
PT	0	0	0	0.005	0.395	**0.561**	0.039	0.000	124	118
PTC / PRP	0.384	**0.594**	0.021	0	0	0	0	0	35	40
PV	**0.908**	0.092	0.000	0	0	0	0	0	26	29
Not Valid^#^	-	-	-	-	-	-	-	-	203	222

* The estimated probabilities of each party/coalition earning more than 7 seats are zero, that’s why the probabilities were omitted from the table.

^#^ Blank, null or missing votes.

Data from Table 1.

Highlighting probabilities indicate the real number of seats received by each party.

A performance rate of 0.715 was obtained to the methodology from a sample of 1,000 voters, where 203 were blank/null votes (as if the sample had only 797 voters) and forecasting the right number of seats (the seat number with greatest probability is the same as the real result) for 15 of 19 parties, which is a good performance (Table 2). By the results, the methodology seems efficient since the major part of greatest probabilities for each party were on the same number of seats as in the real election. Results of some parties diverged from the real, due to the fact that the samples were randomly selected and may not be a good representation of the population. An example is the coalition PSDC/PT do B, which was overrated on the selected sample. Nevertheless, the wrong predictions diverged from the real results by only one seat. In the perfect sample, the performance rate was 0.741, forecasting the right number of seats for 17 of 19 parties.

**Fig 1:**
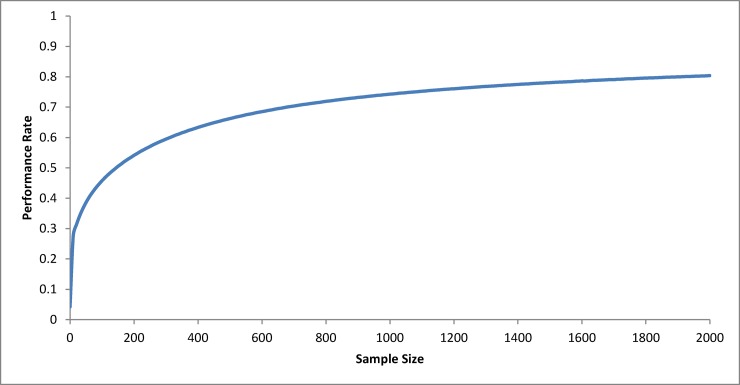
Performance rate for different sizes of perfect samples.

Fig. 1 displays the performance rate of the methodology for different sizes of samples representing the population perfectly. Perfect samples, despite being unlikely on real situations, are the best way to evaluate the performance of the proposed methodology. Samples of size 0 to 2,000 were used, where on sample of size 0, it was assumed that the probability was uniformly distributed among the number of seats, resulting a performance rate of 0.042. As expected, the methodology becomes more efficient when the sample size increases.

**Fig 2:**
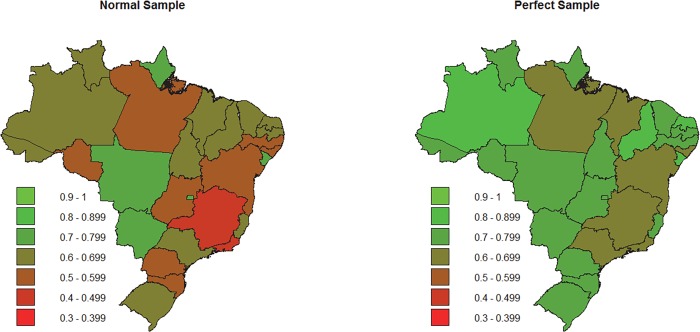
Performance rate for each state using samples of 1,000 voters. Data from the MLA election, Brazil 2010.

Using data from the MLA elections of 2010, simulations were made to each Brazilian state, verifying the performance of the methodology for other states and electoral situations. Table 3 and Fig. 2 present the obtained values for each simulation. A simple random sample and a proportionally perfect sample of size 1,000 were used for each state.

**Table 3:** Performance of the methodology for each state using samples of 1,000 voters.

Election of Members of the Legislative Assembly							
States	Number of parties /coalitions	Performance Rate (Normal Sample)	Performance Rate (Perfect Sample)	Number of Voters	Right prediction* (Normal sample)	Right prediction* (Perfect Sample)	Available Seats
**AC**	10	0.6999	0.7671	470,545	0.8000	0.9000	24
**AL**	8	0.5554	0.6998	2,033,483	0.6250	1.0000	27
**AM**	10	0.6817	0.8018	2,028,122	0.7000	0.9000	24
**AP**	11	0.7461	0.7802	420,331	0.7273	0.9091	24
**BA**	13	0.5983	0.6432	9,544,368	0.7692	0.9231	63
**CE**	15	0.6093	0.7085	5,878,066	0.7333	0.9333	46
**DF**	19	0.7145	0.7414	1,833,942	0.7895	0.9474	24
**ES**	9	0.6529	0.7175	2,521,991	0.6667	0.8889	30
**GO**	14	0.5031	0.7305	4,058,912	0.6429	1.0000	41
**MA**	10	0.6670	0.6959	4,320,748	0.8000	0.9000	42
**MG**	18	0.4411	0.6155	14,513,934	0.3333	1.0000	77
**MS**	7	0.7927	0.7503	1,700,912	1.0000	1.0000	24
**MT**	7	0.7483	0.7705	2,094,032	1.0000	0.8571	24
**PA**	14	0.5859	0.6854	4,763,435	0.7143	1.0000	41
**PB**	11	0.6931	0.7185	2,738,313	0.8182	1.0000	36
**PE**	12	0.5753	0.7134	6,256,213	0.6667	0.9167	49
**PI**	11	0.6696	0.8111	2,261,862	0.7273	1.0000	30
**PR**	13	0.5940	0.7404	7,597,999	0.6154	0.9231	54
**RJ**	20	0.4811	0.6073	11,584,083	0.5000	0.9500	70
**RN**	11	0.6752	0.8225	2,245,115	0.6364	0.9091	24
**RO**	10	0.5992	0.7550	1,078,348	0.6000	0.9000	24
**RR**	11	0.6914	0.7140	271,596	0.7273	0.9091	24
**RS**	15	0.6451	0.7110	8,107,550	0.8667	0.9333	55
**SC**	9	0.5731	0.7009	4,536,718	0.4444	0.8889	40
**SE**	10	0.7756	0.8735	1,425,334	0.8000	1.0000	24
**SP**	20	0.6224	0.6435	30,289,723	0.8000	0.9500	94
**TO**	5	0.6003	0.7961	947,906	0.8000	1.0000	24

Data from the MLA election, Brazil 2010.

* “Right prediction” means the proportion of parties/coalitions which the number of seats with the greatest probability is the same as the real result.

Performance rates of perfect samples were superior to 60% and were superior to 50% in most cases of normal samples. One problem of the performance rate utilized is the devaluation of the result when the probability is greatly distributed among the seats of the party, even when the greatest probability corresponds the real result, because the rate only shows the proportion of the total probability that match with the real result of the election. Column “Right Predictions” from Table 3 shows the proportion of seats where the party’s/coalition number of seats with the greatest probability was the same as the real election. It is possible to verify that even states with low performance rate present high right predictions scores. To perfect samples the proportion of right predictions shows the efficiency of the methodology, being in most cases superior to 90%.

An interesting observation is that the performance rate shows a negative association with the number of parties (Pearson correlation = −0.592; *p* = 0.001), the number of seats (Pearson correlation = −0.775; *p*<0.001) and the number of votes (Pearson correlation = −0.642; *p*<0.001). These results were obtained considering the perfect sample and suggest that scenarios with large number of parties, large number of seats and/or large number of votes, need a larger sample size to get the same performance. Furthermore, we observed no significant correlation (*p*>0.05) between the right prediction index and the number of parties, number of seats and number of votes.

Minas Gerais state (MG) results are interesting because it had a performance rate of 0.441 and 33% of right predictions for the normal sample and, performance rate of 0.616 and 100% of right predictions for the perfect sample. It happened due to a bad sample that influenced the results. Table 4 shows the results from the normal sample and the ones from the perfect sample.

**Table 4:** Samples obtained from election of the MLA in Minas Gerais State. 2010.

Parties/Coalitions	Normal Sample	Perfect Sample	Difference	Parties/Coalitions	Normal Sample	Perfect Sample	DIfference
**PP / DEM / PSDB**	169	158.503	10.497	**PSC**	32	21.936	10.064
**PRB / PT**	131	115.366	15.634	**PC DO B**	16	21.040	-5.040
**PMDB**	82	75.695	6.305	**PTN / PHS**	13	20.534	-7.534
**PTB / PSB**	49	64.509	-15.509	**PR**	10	12.617	-2.617
**PV**	46	53.029	-7.029	**PSOL**	5	2.649	2.351
**PSL / PSDC / PMN**	42	49.437	-7.437	**PSTU**	0	0.645	-0.645
**PDT**	43	44.196	-1.196	**PCB**	0	0.639	-0.639
**PRTB / PTC**	37	32.383	4.617	**PCO**	0	0.114	-0.114
**PPS**	28	26.283	1.717	**Null**	268	277.391	-9.391
**PRP / PT do B**	29	23.034	5.966				

Source: Statistics of Brazilian Superior Electoral Court [5]

In Mato Grosso do Sul state (MS) the performance rate of the normal sample was better than the perfect sample. It happened due to extra information the normal sample had because of a lower number of blank votes when compared with perfect sample (Table 5).

**Table 5:** Samples obtained from election of the MLA in Mato Grosso do Sul State. 2010.

Parties/Coalitions	Normal Sample	Perfect Sample	Difference
**PMDB / PR / DEM / PSDB**	375	365.378	9.622
**PP / PT**	160	160.152	-0.152
**PRB / PPS / PRTB / PHS / PT do B**	95	86.527	8.473
**PDT / PSL / PSDC**	79	75.997	3.003
**PTB / PTN / PMN / PTC / PSB**	48	45.596	2.404
**PSC / PV / PRP / PC DO B**	28	28.257	-0.257
**PSOL**	1	1.459	-0.459
**Blank/Null/Missing**	214	236.635	-22.635

Source: Statistics of Brazilian Superior Electoral Court [5]

Different from what occurred to Minas Gerais state (MG), that also received less null votes on the normal sample, Mato Grosso do Sul state (MS) sample didn’t overestimate or underestimate any party/coalition, it divided the remaining votes proportionally.

### Election of Federal Chamber of Deputies

Results of each state to the elections for the Federal Chamber of Deputies in Brazil are presented in Table 6.

**Table 6:** Performance of the methodology for each state using samples of 1,000 voters. Data from the election of Federal Chamber of Deputies, Brazil 2010.

**Election of Federal Chamber of Deputies**							
States	Number of parties /coalitions	Performance Rate (Normal Sample)	Performance Rate (Perfect Sample)	Number of Voters	Right prediction* (Normal Sample)	Right prediction* (Perfect Sample)	AvailableSeats
**AC**	3	0.9599	0.8208	470,545	1.0000	1.0000	8
**AL**	6	0.9255	0.8285	2,033,483	1.0000	0.8333	9
**AM**	7	0.9997	0.9998	2,028,122	1.0000	1.0000	8
**AP**	6	0.9511	0.9584	420,331	1.0000	1.0000	8
**BA**	10	0.7811	0.7842	9,544,368	1.0000	1.0000	39
**CE**	10	0.9047	0.9169	5,878,066	1.0000	1.0000	22
**DF**	11	0.8750	0.9401	1,833,942	0.9091	1.0000	8
**ES**	5	0.8738	0.9554	2,521,991	1.0000	1.0000	10
**GO**	6	0.9266	0.9131	4,058,912	1.0000	1.0000	17
**MA**	7	0.5569	0.7987	4,320,748	0.4286	1.0000	18
**MG**	13	0.5945	0.7221	14,513,934	0.6154	0.9231	53
**MS**	4	0.8178	0.7850	1,700,912	1.0000	1.0000	8
**MT**	6	0.7135	0.7730	2,094,032	0.6667	0.8333	8
**PA**	7	0.9314	0.8027	4,763,435	1.0000	0.8571	17
**PB**	8	0.9590	0.9709	2,738,313	1.0000	1.0000	12
**PE**	9	0.8876	0.9126	6,256,213	1.0000	1.0000	25
**PI**	9	0.9767	0.9733	2,261,862	1.0000	1.0000	10
**PR**	12	0.8152	0.8505	7,597,999	0.9167	1.0000	30
**RJ**	15	0.5396	0.6470	11,584,083	0.5333	0.9333	46
**RN**	10	0.8973	0.9668	2,245,115	0.9000	1.0000	8
**RO**	5	0.8597	0.8075	1,078,348	1.0000	1.0000	8
**RR**	5	0.7151	0.7901	271,596	0.6000	0.8000	8
**RS**	13	0.7951	0.8078	8,107,550	0.9231	0.9231	31
**SC**	9	0.7832	0.8704	4,536,718	0.7778	0.8889	16
**SE**	8	0.9873	0.9986	1,425,334	1.0000	1.0000	8
**SP**	17	0.5677	0.7051	30,289,723	0.5294	1.0000	70
**TO**	2	0.5560	0.8483	947,906	1.0000	1.0000	8

* “Right predictions” means the proportion of parties/coalitions which the number of seats with the greatest probability is the same as the real result.

Source: Statistics of Brazilian Superior Electoral Court [5]

**Fig 3:**
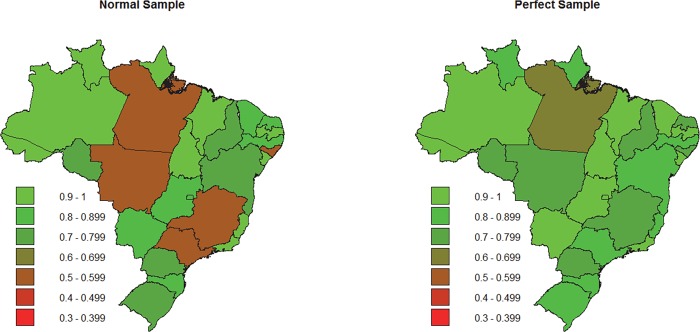
Performance rate for each state using samples of 1,000 voters. Data from the election of Federal Chamber of Deputies, Brazil 2010.

As expected, the performance rates and the proportion of right predictions to the election for the Federal Chamber of Deputies were better than the MLA elections. As previously mentioned, it happened because elections for the Federal Chamber of Deputies have fewer parties and seats than MLA elections. The election for the Federal Chamber of Deputies presented same situations as the MLA, like the performance rate of normal samples better than the perfect sample and the bad performance rate of normal samples due to bad samples. Fig. 3 compares the performance of normal samples with perfect samples by state.

Moreover, the performance rate shows a negative association with the number of seats (Pearson correlation = −0.618; *p*<0.001) and the number of votes (Pearson correlation = −0.547; *p*<0.003) in election for the Federal Chamber of Deputies. Differently from the MLA election, we observed no significant association between the number of parties and the performance rate (Pearson correlation = −0.301; *p*<0.127). Furthermore, we observed no significant correlation (*p*>0.05) between the right prediction index and the number of parties, number of seats and number of votes.

## CONCLUSIONS

Polls for majoritarian voting system usually show estimates of the percentage of votes for each candidate. On proportional systems, estimates of the percentage of votes of each party/coalition do not allow to forecast the number of seats each party/coalition will receive. Thus, classical methods used in majoritarian elections cannot be applied on proportional elections. This paper presented a Bayesian inference on proportional elections considering the Brazilian system of seats distribution, answering the probability that a given party will have representation on the Chamber of Deputies. Results based on data from the Brazilian election for Members of the Legislative Assembly and Federal Chamber of Deputies in 2010 show that most part of the greatest probabilities of each party was concentrated on the number of seats that were equivalent to the real result. Deviations from the real result happened mostly due to the utilized sample, since it might not have been a good representation of the real population. This is spotted when compared to the perfect sample result that presented a good precision estimating the number of seats each party/coalition would receive, with more than 80% of right predictions in all results on both elections. In this context, the success of the inference depends on a sample that should be a good representation of the population.

The proposed methodology is conservative with the indecisive voters. By the partition property of Dirichlet distribution, the indecisive voters do not participate in the analysis. A sample of 1,000 voters of which 200 are indecisive is probabilistically equivalent to a sample of 800 voters with no indecisive voters. This is different than, for example, to distribute (uniformly or proportionally) the indecisive between the parties/coalitions.

The methodology proved to be consistent since it becomes more efficient when the sample size increases. However, states with lots of parties, voters or seats need larger sample size to get the same performance. A suggestion for a future work is a simulation study to define the ideal sample size to obtain, for example, a performance of 90% for all states.

This paper can encourage the use of a Bayesian methodology on proportional elections. To provide a simple, consistent and easily implementable methodology may shorten the distance between Bayesian inference and political researches.

## Supporting Information

S1 Data Data – Election 2010 – Brazil. (XLSX)
